# Update on the Transmission of Zika Virus Through Breast Milk and Breastfeeding: A Systematic Review of the Evidence

**DOI:** 10.3390/v13010123

**Published:** 2021-01-18

**Authors:** Elizabeth Centeno-Tablante, Melisa Medina-Rivera, Julia L. Finkelstein, Heather S. Herman, Pura Rayco-Solon, Maria Nieves Garcia-Casal, Lisa Rogers, Kate Ghezzi-Kopel, Mildred P. Zambrano Leal, Joyce K. Andrade Velasquez, Juan G. Chang Asinc, Juan Pablo Peña-Rosas, Saurabh Mehta

**Affiliations:** 1 Division of Nutritional Sciences, Cornell University, Ithaca, NY 14853, USA; ect53@cornell.edu (E.C.-T.); mm2463@cornell.edu (M.M.-R.); jfinkelstein@cornell.edu (J.L.F.); hsh35@cornell.edu (H.S.H.); 2 Department of Maternal, Newborn, Child and Adolescent Health and Ageing, World Health Organization, Geneva CH-1211, Switzerland; raycosolonp@who.int; 3 Department of Nutrition and Food Safety, World Health Organization, Geneva, CH-1211, Switzerland; garciacasalm@who.int (M.N.G.-C.); rogersl@who.int (L.R.); penarosasj@who.int (J.P.P.-R.); 4 Albert R. Mann Library, Cornell University, Ithaca, NY 14853, USA; kwg37@cornell.edu; 5 Hospital de Niños Roberto Gilbert Elizalde, Guayaquil 090514, Ecuador; mzambranol@jbgye.org.ec (M.P.Z.L.); jandradev@jbgye.org.ec (J.K.A.V.); jgca76@yahoo.com (J.G.C.A.)

**Keywords:** Zika virus, Zika virus infection, perinatal transmission, mother-to-child transmission, breast milk, breastfeeding

## Abstract

We systematically searched regional and international databases and screened 1658 non-duplicate records describing women with suspected or confirmed ZIKV infection, intending to breastfeed or give breast milk to an infant to examine the potential of mother-to-child transmission of Zika virus (ZIKV) through breast milk or breastfeeding-related practices. Fourteen studies met our inclusion criteria and inform this analysis. These studies reported on 97 mother–children pairs who provided breast milk for ZIKV assessment. Seventeen breast milk samples from different women were found positive for ZIKV via RT-PCR, and ZIKV replication was found in cell cultures from five out of seven breast milk samples from different women. Only three out of six infants who had ZIKV infection were breastfed, no evidence of clinical complications was found to be associated with ZIKV RNA in breast milk. This review updates our previous report by including 12 new articles, in which we found no evidence of ZIKV mother-to-child transmission through breast milk intake or breastfeeding. As the certainty of the present evidence is low, additional studies are still warranted to determine if ZIKV can be transmitted through breastfeeding.

## 1. Introduction

Zika virus (ZIKV), a virus from the *Flaviviridae* family, is an arthropod-borne virus. Local ZIKV transmission has been reported in 87 countries and territories, including the Americas, Asia, Africa, and the Western-Pacific region [1]. ZIKV infection during pregnancy can lead to congenital Zika syndrome, which is characterized by severe central nervous system malformations in developing fetuses such as congenital microcephaly [2,3,4,5]. Several ZIKV outbreaks have occurred worldwide, most predominantly in the Western Hemisphere; including outbreaks in Yap Island in 2007 [6], French Polynesia in 2013 [7], and most recently, Brazil and the Americas, with an exponential increase in cases between 2014 and 2016 [8]. Due to the unprecedented rise in ZIKV cases and the associated risks of pregnancy complications and birth defects, the World Health Organization (WHO) declared the ZIKV outbreak an international public health emergency in November 2016 [9,10,11,12]. In 2018, the WHO included ZIKV infection in the Research and Development Blueprint list among the priority diseases that pose the greatest public health risk due to their epidemic potential [13]. 

ZIKV is primarily transmitted via mosquito vectors from the *Aedes* genus, primarily *Aedes aegypti*, the same mosquito that transmits dengue, chikungunya, and yellow fever viruses [12]. Sexual transmission also has been identified [14], and ZIKV RNA has been found in amniotic fluid, breast milk, semen, saliva, urine, and blood [12,15,16]. After exposure, the incubation period is estimated to range from 3 to 14 days, and it is often followed by either an asymptomatic or mild non-specific disease. If symptomatic, clinical manifestations include fever, pruritic maculopapular rash, arthralgia, and headache [16]. To a lesser frequency, other symptoms might include myalgia, gastrointestinal distress, retroorbital pain, and lymphadenopathy. 

Due to the similarity of symptoms with other arbovirus infections and nonspecific clinical presentation or the absence of clinical manifestations, ZIKV infections are often misdiagnosed as other arboviruses infections (e.g., dengue fever), as well as other infections endemic to tropical regions. To facilitate the diagnosis of ZIKV disease, in 2016, the WHO established interim guidance for ZIKV laboratory testing [17]. In this guideline the WHO recommends the collection of whole blood or urine samples for nucleic acid testing, via reverse transcription-polymerase chain reaction (RT-PCR), within the first seven days of symptoms onset. After seven days, viremia drops rapidly, at which point serology and/or RT-PCR are recommended for assessment. 

Despite health systems’ efforts to screen and counsel pregnant women for the potential risks associated with ZIKV infections, the virus remains a major challenge for maternal and child health. ZIKV infection during pregnancy has been associated with intrauterine fetal demise and miscarriage [16,18,19]. Moreover, an estimated 5–15% of infants born to mothers with ZIKV infection have been reported to have congenital complications including microcephaly and a series of congenital malformations referred to as congenital Zika syndrome (CZS) [12,19]. CZS includes microcephaly, brain damage, subcortical calcifications, and a multitude of developmental disorders resulting in pulmonary, ocular, and musculoskeletal defects [20,21,22]. 

While it has been established that mother-to-child transmission of ZIKV may occur during pregnancy or at the time of birth, less is known about transmission through breast milk and breastfeeding practices [23]. In our previous rapid systematic review [15], we found limited evidence of the risk of ZIKV transmission through breast milk intake or breastfeeding. At present, WHO guidelines advise standard breastfeeding practice for all mothers regardless of ZIKV infection status [24]. The current review aims to assess the available evidence of the possible transmission of ZIKV through breast milk or breastfeeding practices to update findings from the initial systematic review, and to contribute to the development of evidence-informed guidelines at a national, regional, and global level.

## 2. Methods

### 2.1. Study Criteria

#### 2.1.1. Types of Studies

We aimed to include the following study designs in this review: randomized controlled trials (RCTs), quasi-RCTs, and all observational studies (i.e., cohort studies, case reports, and surveillance reports). 

#### 2.1.2. Participants

Included participants were breastfeeding women and children with confirmed, probable, or suspected ZIKV infection. This includes participants who were currently breastfeeding, as well as those who were breastfeeding before a ZIKV presumptive diagnosis. Briefly, cases were defined as suspected cases: any infant or breastfeeding woman who had been vaccinated for ZIKV, traveled or lived in an endemic area within the last seven days from the start of symptoms; probable case: a suspected case with a presence of viral antibodies against ZIKV; a confirmed case: any individual with laboratory confirmation of recent ZIKV infection defined by the presence of ZIKV RNA or antigen in serum or other biological samples or IgM antibody against ZIKV positive and plaque reduction neutralization test ≥ 90% (PRNT90) for ZIKV with titer ≥ 20 and ZIKV PRNT90 titer ratio ≥ 4 compared to other flaviviruses. Studies with populations that did not meet these criteria, tested breast milk samples, or had a non-ZIKV infection, were excluded. 

#### 2.1.3. Types of Exposure 

Exposure criteria were described as any woman with ZIKV infection who was breastfeeding or was intending to breastfeed an infant aged from 0 to 2 years.

#### 2.1.4. Types of Outcomes

Primary outcomes included infants with suspected, probable, or confirmed ZIKV infection within 30 days of breastfeeding or receiving expressed breast milk from a woman with suspected, probable, or confirmed infection. Secondary outcomes included detection of ZIKV in breast milk, maternal blood, sweat, or saliva, or infant’s saliva by detection methods that identify suspected, probable, and confirmed cases. Detection methods of ZIKV infection in maternal and infant samples include ZIKV RNA by RT-PCR, ZIKV-specific IgM antibody by ELISA, PRNT90 for ZIKV with titer > 20, and ZIKV PRNT90 titer ratio > 4 compared to other flaviviruses and, ZIKV isolation in culture. 

### 2.2. Search Strategy

A search strategy was designed to identify all relevant evidence, without date or language restriction, pertaining to the possible transmission of ZIKV through breast milk and breastfeeding. The search was adapted from our previous systematic review [15]. An initial search was conducted on 1 May 2019, and an updated search of all databases was conducted on 18 June 2020.

Search terms included variations and permutations of United States National Library of Medicine Medical Subject Headings terms and text words related to infectious agents, breastfeeding, transmission fluids (e.g., breast milk, blood, and sweat), and participants (mother and child). Report characteristics included a time range of all years, any language, and any publication status. An overview of the search strategy is provided as supplementary material.

The following international electronic databases were searched: MEDLINE (PubMed), EMBASE, Cochrane Library, Web of Science (both the Social Science Citation Index and the Science Citation Index), CINAHL, and BIOSIS. The following regional electronic databases were searched: IBECS, Scielo, Global Index Medicus—IMEMR (EMRO), AIM (Africa), LILACS (Americas), IMSEAR (South-East Asia), WPRIM (Western Pacific)), and Native Health Research Database.

### 2.3. Data Extraction and Management:

All included reports were screened independently by two authors using the Covidence systematic review software (Veritas Health Innovation, Melbourne, Australia) every disagreement was resolved by consensus or a third author. A data extraction form was developed and piloted for data extraction. Two authors extracted data and discrepancies were resolved by discussion. It was not possible to calculate effect estimates since all the included studies were observational, case reports, and longitudinal studies, with a limited number of cases.

### 2.4. Quality of the Evidence

The GRADE approach was used to ascertain the certainty of the evidence [25]. Data on the primary and secondary outcomes were considered; ref. [1] ZIKV infection in infants breastfeeding from a mother with confirmed, probable, or suspected ZIKV infection, and [2] the detection of ZIKV RNA in breast milk samples from mothers with confirmed or suspected ZIKV infection. The GRADE approach included the risk of bias, the directness of evidence, inconsistency (heterogeneity), the precision of effect estimates, and the risk of publication bias across the included studies. All the included studies are observational and with few cases, and they were downgraded one level to low-certainty of the evidence, and further study limitations led to downgrading to very-low certainty of the evidence. Considering that all included studies are observational and with few events, the evidence provided by these studies is heterogenous, and they do not allow for pooled estimates, the certainty of the evidence is described as a narrative.

## 3. Results

### 3.1. Study Designs

**Figure 1:**
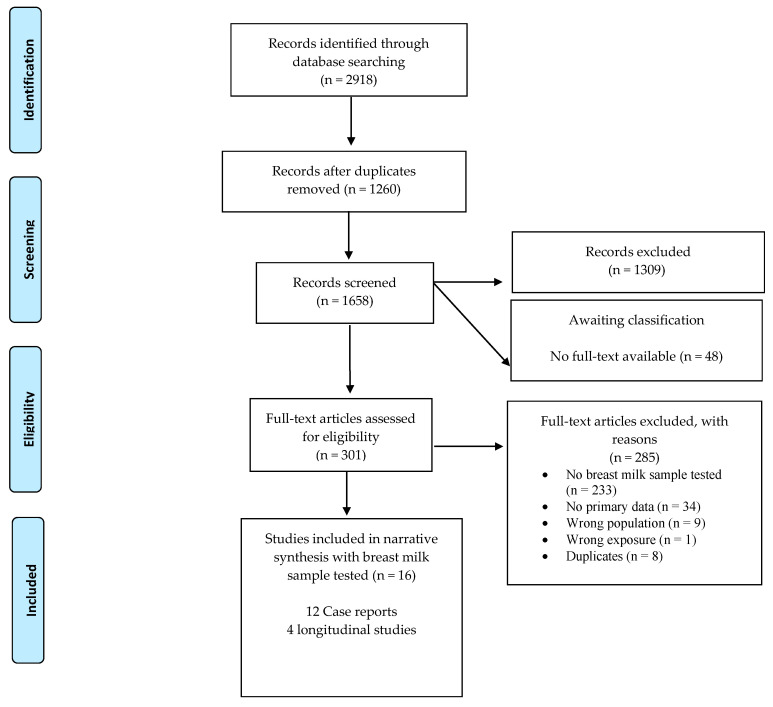
A schematic representation of the search strategy and selection of included studie.

The search strategy identified 2918 records, of which a total of 1658 titles and abstracts were screened. There were 349 full-text articles assessed for eligibility. Among the screened records, only 16 articles met our inclusion criteria (Figure 1) [10,26,27,28,29,30,31,32,33,34,35,36,37,38,39,40]. We did not identify any trials reporting on the assessment of breast milk samples after vaccination for ZIKV infection. Two out of the 16 identified studies [10,27] are also described in our 2017 systematic review [15]. Noteworthy, this updated systematic search identified four different articles that reported on the same cases: Besnard, 2014 [10] and Besnard, 2017 [26] reported on the same two mother and child pairs; similarly, Blohm, 2017 [31] and Blohm, 2018 [32] reported on a single mother and child pair. Only one of each duplicated study was considered during data extraction and analysis.

Among the studies informing our analysis, 10 were case reports [10,26,27,28,29,30,31,32,34,37,39,40], and four were longitudinal studies [33,35,36,38]. The records informing the present analysis included a total of 177 mother and child pairs, but only 97 women provided a breast milk sample for ZIKV assessment. Our data extraction, synthesis of evidence, and analysis are solely based on the maternal–infant pairs with breast milk samples, all other cases were excluded.

### 3.2. Settings

The studies informing our analysis were from Brazil (n = 6) [28,29,30,36,37,38], Colombia (n = 1) [39], France (n = 2) [27,40], French Polynesia (n = 1) [10], Thailand (n = 2) [33,34], Venezuela (n = 1) [32], and Spain (n = 1) [35].

### 3.3. Participants

Among the case reports, there were 14 children assessed for ZIKV infection. Among them, eight neonates tested at birth [28,29,30,37,39,40] or during the first three days of life [10,27]. Additionally, six infants were tested for ZIKV infection between 5 and 11 months of age [28,32,34].

In one longitudinal study [35], 72 pregnant women confirmed or suspected of ZIKV infection were followed until delivery to evaluate potential adverse pregnancy outcomes. Only 15 out of the 72 women provided a breast milk sample for ZIKV assessment within 24 h after delivery. A different longitudinal study [36] aimed to assess the impact of ZIKV infection on breast milk viscosity. Forty pregnant women were recruited, 20 women with confirmed ZIKV infection via RT-PCR assessment, and the remaining 20 women were described as clinically healthy. Women from both groups provided one breast milk sample between 48 and 72 h postpartum [36]. The latter is the only study that included a control group during their assessment. In another study [38], people 16 years and older were recruited from two health centers if they presented with two or more symptoms associated with ZIKV infection in 14 days or less. Among the participants with ZIKV infection, there were a total of 18 pregnant women, of which seven were confirmed by RT-PCR analysis in plasma, serum, or urine. From this study, only one woman provided three breast milk samples for analysis. Another longitudinal study [33], evaluated ZIKV infection in 27 postpartum women. Limited data were available from this cohort in the form of an abstract.

### 3.4. Child Outcomes

There were 97 mother–children pairs included in this analysis who provided breast milk for ZIKV analysis. From these mother and child dyads, the outcomes for 14 cases were described as case reports [10,27,28,29,30,32,34,37,39,40] and 83 pairs in longitudinal studies [33,35,36,38]. In total, this analysis included six infants with ZIKV infection confirmed by viral RNA detection and 50 children with negative RT-PCR tests for ZIKV infection. A summary description of these cases is included in Table 1, and further information is provided in the Supplementary File S3.

Six out of 14 infants from case reports were confirmed with ZIKV infection through viral RNA detection with an RT-PCR assay, five cases tested positive in a blood sample by RT-PCR assay [10,30,32,39], one case was found negative in a cord blood sample but positive in urine by RT-PCR test [29]. One neonate had ambiguous results from the RT-PCR assay [27]. Additionally, saliva samples from three neonates were assessed by RT-PCR [26,37,38], ZIKV RNA was detected in the saliva and blood from one case [26] whose mother also had positive saliva and blood samples. Most mothers described in the case reports had a confirmed ZIKV infection by RT-PCR test, except one mother with negative RNA detection in plasma but positive IgM levels detected [31], another woman who presented symptoms during the first trimester and elevated IgM levels were found at 38th gestational weeks [30]. One pregnant woman had negative RT-PCR and antibody results, as well as her newborn; however, ZIKV RNA was detected in her breast milk [37].

None of the longitudinal studies described children with positive RT-PCR results and all mothers had confirmed ZIKV infection by RNA detection. However, of the nine maternal cases with confirmed ZIKV infection reported by Rodo and colleagues [35], there was one spontaneous abortion, one elective termination of pregnancy due to fetal abnormalities, and one baby was born with brain malformations. In the latter case, the neonate had elevated IgG levels for 24 months after birth. De Quental and colleagues [36] reported on 20 pregnant women with confirmed ZIKV infection and 20 pregnant women with no ZIKV infection; there were no cases of microcephaly at birth in either group. However, during the subsequent 12 months, six women who had ZIKV infection during pregnancy reported neurological complications in their infants, these included convulsions, hearing, and vision impairments, and neuropsychomotor developmental delay. Diagnostic tests were not reported for any infant included in this study [36]. Buathong and collaborators [33] reported outcomes of the six neonates whose mothers had a positive breast milk sample. These six cases had negative RT-PCR and IgM tests for ZIKV infection and did not present signs of CZS at birth. One infant reported by Tozetto-Mendoza and colleagues [38] tested negative in cord blood, saliva, and urine samples analyzed by RT-PCR.

### 3.5. Zika Virus in Breast Milk

Of the 97-breast milk from different women assessed for ZIKV, only 17 breast milk samples were positive for ZIKV RNA by RT-PCR. No Zika viral RNA was detected in the remaining 80 breast milk samples. Ten of the positive breast milk samples were described in case reports [10,27,28,29,30,32,34,37,39], and seven cases in longitudinal studies [33,38]. Among case reports of children putatively exposed to positive breast milk samples, there were five neonates [10,29,30,39] and one five-month-old infant [32] with confirmed ZIKV infection by RT-PCR test, and one neonate [27] with ambiguous RT-PCR results. These cases are briefly described in Table 1 and more information can be found in the Supplementary File S3.

Of the studies with positive breast milk samples, there is no information on the number of samples collected from the six women reported by Buathong and colleagues [33]. In another study, three breast milk samples were collected from one woman and were assessed by RT-PCR; one sample was positive at 20 days after onset of maternal symptoms and the two other samples, collected at 23 and 30 days after onset of symptoms, were negative [38].

Three breast milk samples from one woman were positive for ZIKV RNA; all were collected 14 days after maternal symptoms onset during the 36th gestational week. Two subsequent samples were collected from the same mother, the second and third samples were collected two weeks and nine days after the first sample [29]. All samples were positive for viral RNA. Another 13 positive breast milk samples from different women were tested from 2 days to 2 weeks after maternal symptom onset [10,27,28,30,32,33]. In another woman, a breast milk sample, with detected viral RNA was tested at birth, 30 days after the onset of maternal symptoms [39]. Another positive ZIKV RNA breast milk sample was collected after birth from an asymptomatic woman who had a baby with severe microcephaly 19 months earlier from a previous pregnancy [37]. In one study, analysis of stored breast milk samples from three days prior to onset of maternal symptoms [34] detected presence of ZIKV RNA.

Viral cell culture was attempted from seven breast milk samples corresponding to seven different women [10,27,28,29,32], out of which ZIKV replication was detected in cell cultures from five breast milk samples [27,28,29,32,38]. Notably, one case report found that viral RNA isolated from breast milk and child serum were genetically related to each other [30]. Another case report found identical viral isolates from mother and child based on the NS5 gene sequence, and 99% identity with two nucleotide substitutions in full-genome sequencing of ZIKV isolates from breast milk and child’s urine [32]. A different study [33] identified the Asian lineage from ZIKV isolates in breast milk, confirming the presence of ZIKV.

For the breast milk samples with negative RT-PCR tests for ZIKV RNA, in the case of two infants, the breast milk samples were tested within 10 days of maternal symptoms onset [28], whereas in another two cases [28,40] the breast milk samples were collected between 4 and 6 months after the onset of maternal symptoms. In the two longitudinal studies [35,36], maternal ZIKV infection occurred mostly during the first and second trimesters of pregnancy.

The search strategy did no identify any studies assessing ZIKV in breast milk after vaccination. Furthermore, there were no studies evaluating the presence of ZIKV in maternal sweat, tears, or skin among the mother-infant dyads providing breast milk samples.

### 3.6. Infant Feeding Practices

Among cases with a positive breast milk sample for viral RNA and confirmed infant infection, there was one three-day-old neonate who was breastfed since birth and one four-day-old-neonate who was fed both breast milk and infant formula [10]. One newborn was formula fed [29], and one five-month-old infant with confirmed ZIKV infection was breastfed during the symptomatic maternal phase [32]. Infant feeding practices were not reported in two newborn cases.

There were 10 cases where a breast milk sample tested positive for viral RNA, but the infant had no infection. One exclusively breastfed newborn was tested at birth and evaluated for seven months of follow-up [37], while six other newborns were weaned when breast milk tested positive [33]. In another 10-month old infant, breastfeeding was interrupted seven days after maternal symptoms onset and restarted four days after symptoms resolved [28]. Another 10-month old infant was exclusively breastfed until the viral RNA was detected in maternal serum and breast milk samples; however, viral RNA was detected in 11 stored breast milk samples from three days before breastfeeding cessation [34]. Infant feeding practices were not reported in one newborn [38].

### 3.7. Certainty of the Evidence

The certainty of the evidence was assessed following the GRADE approach, and all the studies were of very low certainty. All studies were observational, and only a limited number tested breast milk samples. Further, the studies lacked control groups, and there was incomplete reporting on ZIKV infection among infants by molecular or serological tests. Infant feeding practices were scarcely reported, and there was limited information about exclusive breastfeeding either by feeding at the breast or with expressed milk. Furthermore, the authors did not provide any information about skin-to-skin contact, rooming, and other commonly employed breastfeeding practices.

## 4. Discussion

This review identified 97 mother–infant pairs providing breast milk samples for analysis. Of the six infants with confirmed ZIKV infection by RT-PCR test in blood or urine samples, only three children were reported to be breastfed. Moreover, five positive cases were diagnosed at birth or shortly after, suggesting that infection could have occurred during pregnancy, delivery, or the perinatal period. Given that other potential external routes of transmission were not considered, it was not possible to fully exclude ZIKV transmission through mosquito bites or contact with blood, saliva, or exposure to other maternal bodily fluids. Only three of the included studies assessed saliva samples [10,37,38]; in one case, both maternal and infant saliva samples were positive for ZIKV, suggesting that saliva could also be a potential transmission route between mothers and infants.

To evaluate if the presence of the viral particles in breast milk might be infectious and potentially hazardous to the breastfed infant, viral culturing is recommended. Only five out of the seventeen breast milk samples from different women were assessed by viral culturing. These reports successfully cultured the ZIKV from breast milk isolates, suggesting that breast milk is a potential route of exposure to breastfed children. However, while it is well documented that viral infection during pregnancy results in congenital defects, the consequences of exposure to positive breast milk and viral infection during infancy or early childhood have not been fully elucidated.

The transmission of ZIKV by breast milk intake or breastfeeding could be affected by several factors including maternal viral load, milk composition, and infant feeding practices. Among the different viruses that have been detected in breast milk, cytomegalovirus was found in the breast milk of seropositive mothers with higher viral DNA at four to six weeks postpartum and the lowest levels found in colostrum [41]. This suggests that viral kinetics could change during the lactation period, and the assessment of longitudinal samples might be needed to fully understand viral dynamics. The human immunodeficiency virus (HIV) has been extensively studied in the context of mother-to-child transmission, the risk of HIV transmission by breast milk intake is estimated to be 0.74% per month of breastfeeding in the absence of antiretroviral treatment [42]. Moreover, the risk of HIV transmission has been reported to be higher among infants mix-fed with breast milk and breast milk substitutes in comparison with exclusively breastfed children [43]. Most of the studies included in this review assessed breast milk samples obtained shortly after birth, without serial samples over time and sparse reporting of infant feeding practices. It will be critical to consider these aspects to further understand the dynamics of possible viral transmission by breast milk intake. Additionally, future studies should consider the possible coinfection with dengue virus or chikungunya virus, as these viruses are transmitted by the same mosquito vector as ZIKV. Co-infection could affect disease progression, and in some cases increase disease severity [44] which could in turn affect the viral dynamics in breast milk. Although none of the mothers or children included in this review reported co-infections, diagnostic results for dengue or chikungunya viruses were not reported in all studies.

There are three previously published systematic reviews assessing breast milk as a potential route of ZIKV transmission. The first [15] was a rapid systematic review, was undertaken by our group, and described two studies reporting on three cases of ZIKV-infected breastfeeding mothers. ZIKV RNA in the breast milk samples from the three women and two of the newborns were positive for ZIKV infection. In a different systematic review, the authors investigated flavivirus transmission through breast milk [45] and found five articles relevant to ZIKV transmission. Another systematic review [46] examining the possible transmission of ZIKV through breast milk, identified ten mother-and-child pairs, and found no evidence of long-term child complications. 

The current review expands the scope of the literature search and consequently the number of mother–child pairs included in these analyses. In this updated review, we synthesized all the evidence available, including the two reports assessed in our previous review [15] and of 12 additional reports, of which eight articles have not been described elsewhere. Similar to previous publications, although there is evidence of ZIKV RNA presence in breast milk, there is no clear evidence of disease or clinical complications in infants that could be associated with the intake of ZIKV positive breast milk or breastfeeding practices. Additionally, the present review aimed to assess the possible risk of ZIKV transmission by other body fluids such as sweat, saliva, and tears which could be involved in viral transmission during breastfeeding. Only three studies assessed saliva samples but none in tears or sweat.

Limitations

The findings from this review are limited by the lack of studies regarding mother-to-child transmission through breast milk intake and breastfeeding, the lack of concurrent assessment of other potentially infective maternal body fluids, and limited description and follow-up of cases. Among the cases reported, it was not possible to discern if infant infection occurred in utero, during labor, by contact with saliva, sweat, or skin, or if it was the result of vector borne transmission, particularly considering that the majority were reported in endemic areas. There were few longitudinal studies identified, and of those included, most did not report longitudinal data on breast milk sample collection and analysis. Most of the evidence of breast milk infection was provided by case reports, which were limited by small sample size, lack of serial breast milk samples, short follow-up period, and no control groups. This increases the imprecision and uncertainty of the results. Particularly, the lack of serial breast milk samples limits the understanding of viral dynamics and the short follow-up period prevents the assessment of possible long-term consequences of viral exposure among mothers and infants. Moreover, the heterogeneity among the study designs prevented pooling results to calculate risk estimates. 

There is a high risk of publication and sample bias among these reports, given that cases with detected ZIKV RNA in breast milk or children are most likely to be reported, and pregnant women or infants with more severe symptoms are most likely to seek health care and receive a diagnostic test.

## 5. Conclusions

More evidence is needed to understand the possible risk of ZIKV transmission through breastfeeding. It is essential to assess infant cases, where viral exposure may not have occurred in utero, with concurrent evaluation of maternal viremia, breast milk viral load and composition, especially for ZIKV antibodies that may confer passive immunity to the child. The assessment of serial breast milk samples from women with ZIKV virus and consistent reporting of the methods for obtaining and processing breast milk samples will be critical to understanding viral dynamics in breast milk and the potential of viral transmission. Additionally, as ZIKV RNA has been detected in the skin [47], saliva [48], and conjunctival fluids [49,50], suggesting potential transmission routes due to increased contact between mother and the child while caring and breastfeeding, it is important to consider the assessment of these bodily fluids when evaluating the risk of mother-to-child transmission of ZIKV through breastfeeding.

In summary, this systematic review included evidence from 10 case reports and four longitudinal studies. There were 97 mother–infant pairs from where a breast milk sample was available for analysis; 77 were cases where the mother had suspected or confirmed ZIKV infection. Six children were confirmed to have ZIKV infection by RT-PCR, and in all six cases, a breast milk sample was positive for viral RNA. There were 60 other breast milk samples without detectable levels of viral RNA. Considering the evidence, the review authors are uncertain of the risk of child infection through breast milk intake or breastfeeding from a woman with ZIKV infection and determined the certainty of the evidence as very low and identified several research gaps.

## Figures and Tables

**Table 1:** Characteristics of mother and infant pairs included in the narrative analysis.

		Children Outcomes			Breast Milk Outcomes			Maternal Outcomes		
Study	Design	Children with ZIKV Infection	Blood RT-PCR	Infant Feeding Practices	RT-PCR	Culture	Viral RNA Sequencing	Confirmed ZIKV Infection	Diagnostic Essay	Country
Besnard 2014 [10] Besnard 2017 [26]	Case reports	Yes	Positive in blood saliva	Breastfeeding	Positive	Negative	NA	Yes	Positive RT-PCR, 5 days after symptoms onset	French Polynesia
		Yes	Positive	Not clear	Positive	Negative	NA	Yes	Positive RT-PCR, 2 days after symptoms onset	
Dupont 2016 [27]	Case report	Undetermined	Ambiguous	Breastfeeding	Positive	Positive	NA	Yes	Positive 3 days after symptoms onset	New Caledonia
Blohm 2017 [31] Blohm 2018 [32]	Case report	Yes	Plasma positive	Breastfed for 5 months	Positive	Positive	99% identity with the virus isolated from the child’s urine	Yes	Negative RT-PCR, 5 days after symptoms onsetPositive for IgM and marginal IgG	Venezuela
Cavalcanti 2017 [28]	Case reports	No	Serum, negative	Mix-feeding	Positive	Positive	NA	Yes	Positive RT-PCR	Brazil
		No	NA	Breastfeeding	Negative	NA	NA	Yes	Positive RT-PCR	
		No	Serum, Negative for ZIKV Serum, Positive for CHIKV	Breastfeeding	Negative	NA	NA	Yes	Positive RT-PCR	
		No	Serum, negative	Breastfeeding	Negative	NA	NA	Yes	Positive RT-PCR	
Sotelo 2017 [29]	Case report	Yes	Cord blood, negative	Not reported	Positive for samples collected at 37th gestational week, at birth, 38th gestational week, and 10th weeks postpartum	Positive for colostrum and sample 10 days after birth	NA	Yes	Positive RT-PCRIgM and IgG positive	Brazil
Giovanetti 2018 [30]	Case report	Yes	Serum, positive	Not reported	Positive	NA	Positive for strain similarities found with newborn viral genome sequencing obtained from the newborn	Yes	IgM positive. IgG not reported	Brazil
Desclaux 2018 [40]	Case report	No	Negative (serum)	Not reported	Negative	NA	NA	Yes	Positive RT-PCR	France
Mello 2018 [37]	Case report	No	Negative in blood and saliva	Breastfeeding	Positive	NA	NA	No	Negative RT-PCR and Antibodies	Brazil
Villamil-Gomez 2017 [39]	Case report	Yes	Positive (serum) at birth and 4, 6 months	Not reported	Positive	NA	NA	Yes	Positive RT-PCR at birth and 4, 6 months	Colombia
Hemachudha 2019 [34]	Case report	No	NA	Breastfeeding interrupted due to maternal diagnosis	Positive, starting 3 days before maternal symptoms and remained positive for 11 days (22 samples total).	NA	NA	Yes	Positive RT-PCR	Thailand
Buathong 2017(Abstract only) [33]	Cohort	No 9 from confirmed cases and 62 from probable cases	Negative	Breastfeeding interrupted due to maternal diagnosis	Positive	NA	Asian lineage identified	Yes, all 6 women	Positive (method unclear)	Thailand
Rodo 2019 [35]	Cohort	NA	Negative (serum)	Not reported	Negative	NA	NA	72 of 254 women were positive	Positive (9 cases) by RT-PCR 62/71, 87.3%, positive for ZIKV IgM and/or ZIKV IgG and positive. 4 cases had positive IgM, 3 cases had positive both IgM and RT-PCR tests. 9 cases had detectable IgG levels.	Spain
De Quental 2019 [36]	Cohort	No	NA	Not reported	Negative	NA	NA	Yes, 20 women were infected	Positive (20 cases) by RT-PCR	Brazil
Tozetto-Mendoza 2019 [38]	Cohort	No	Negative cord blood, saliva, and urine.	Not reported	Positive	Positive	NA	Yes, 1	Positive (94/235) confirmed ZIKV infection by RT-PCR in either plasma, serum, or urine. At least 7 positive cases were pregnant women.	Brazil

## Data Availability

All the included data is from studies that have been cited in the review and listed in the references section.

## Supplementary Materials

The following are available online at https://www.mdpi.com/1999-4915/13/1/123/s1, File S1, Checklist-PRISMA Checklist, File S2, Search strategy, File S3, Table-Studies description.
