# Supplementary material for: Update on the Transmission of Zika Virus Through Breast Milk and Breastfeeding: A Systematic Review of the Evidence
Source: Viruses. 2021 Jan 18;13(1):123. doi: 10.3390/v13010123 (PMC7830280; doi:10.3390/v13010123)
Supplement: Supplementary file 1 [file viruses-13-00123-s001.zip › viruses-1047543-SI/File S2-Search strategy overview.docx]

**Update on the transmission of Zika virus through breast milk and breastfeeding: A systematic review of the evidence**

**Search Strategy Overview**

**MEDLINE (PubMed)**

1. (Zika virus[Mesh] OR Zika virus infection[Mesh] OR Zika[tiab] OR ZikV[tiab])
2. (Sweat[MeSH] OR Blood[MeSH] OR Mucus[MeSH] OR Saliva[MeSH] OR Tears [MeSH] OR Infectious Disease Transmission, Vertical[MeSH] OR sweat*[tiab] OR blood*[tiab] OR mucus[tiab] OR serum[tiab] OR sera[tiab] OR fluid*[tiab] OR saliva[tiab] OR tears[tiab] OR vertical transmission[tiab] OR postnatal transmission[tiab] OR post natal transmission[tiab] OR maternal-infant transmission[tiab] OR adult-to-child[tiab] OR maternal-to-child[tiab] OR mother-to-child[tiab] OR MTCT[tiab] OR PMTCT[tiab])
3. (Breast Feeding[Mesh] OR Milk, Human[Mesh] OR Lactation[Mesh] OR colostrum[mesh] OR Breastfe*[tiab] OR breast fe*[tiab] OR breastmilk[tiab] OR breast milk[tiab] OR human milk[tiab] OR maternal milk[tiab] OR prelacteal feed*[tiab] OR lactati*[tiab] OR colostrum[tiab] OR mixed feeding[tiab] OR mother’s milk[tiab] OR expressed milk[tiab] OR milk bank*[tiab])
4. (Pregnant Women [Mesh] OR Pregnancy [Mesh] OR Prenatal Care [Mesh] OR Mothers[Mesh] OR infant[MeSH] OR pregnan*[tiab] OR gestat*[tiab] OR perinatal[tiab] OR antenatal[tiab] OR parturi*[tiab] OR mother*[tiab] OR infant*[tiab] OR maternal[tiab] OR baby[tiab] OR babies[tiab] OR newborn*[tiab] OR neonat*[tiab])
5. #4 AND #2
6. #3 AND #1
7. #5 AND #1
8. #6 OR #7
